# Mathematical Modeling of the Pituitary–Thyroid Feedback Loop: Role of a TSH-T_3_-Shunt and Sensitivity Analysis

**DOI:** 10.3389/fendo.2018.00091

**Published:** 2018-03-21

**Authors:** Julian Berberich, Johannes W. Dietrich, Rudolf Hoermann, Matthias A. Müller

**Affiliations:** ^1^Institute for Systems Theory and Automatic Control, University of Stuttgart, Stuttgart, Germany; ^2^Medical Department I, Endocrinology and Diabetology, Bergmannsheil University Hospitals, Ruhr University of Bochum, Bochum, Germany; ^3^Ruhr Center for Rare Diseases (CeSER), Ruhr University of Bochum, Bochum, Germany; ^4^Ruhr Center for Rare Diseases (CeSER), Witten/Herdecke University, Bochum, Germany; ^5^Private Consultancy Research & Development, Yandina, QLD, Australia

**Keywords:** thyroid hormones, pituitary–thyroid feedback loop, mathematical modeling, diagnosis, TSH-T_3_-shunt, sensitivity analysis

## Abstract

Despite significant progress in assay technology, diagnosis of functional thyroid disorders may still be a challenge, as illustrated by the vague upper limit of the reference range for serum thyrotropin (*TSH*). Diagnostical problems also apply to subjects affected by syndrome T, i.e., those 10% of hypothyroid patients who continue to suffer from poor quality of life despite normal *TSH* concentrations under substitution therapy with levothyroxine (*L*-*T*_4_). In this paper, we extend a mathematical model of the pituitary–thyroid feedback loop in order to improve the understanding of thyroid hormone homeostasis. In particular, we incorporate a *TSH*-*T*_3_-shunt inside the thyroid, whose existence has recently been demonstrated in several clinical studies. The resulting extended model shows good accordance with various clinical observations, such as a circadian rhythm in free peripheral triiodothyronine (*FT*_3_). Furthermore, we perform a sensitivity analysis of the derived model, revealing the dependence of *TSH* and hormone concentrations on different system parameters. The results have implications for clinical interpretation of thyroid tests, e.g., in the differential diagnosis of subclinical hypothyroidism.

## 1 Introduction

In recent years, the mathematical modeling of human thyroid hormone homeostasis via the hypothalamic–pituitary–thyroid feedback loop has received an increasing amount of attention. Starting from early phenomenological models, more precise models have been developed based on molecular and pharmacokinetic data, see, e.g., Ref. (1–3, 4–6) for recent surveys on existing modeling approaches. These mathematical models can give important insight into the functionality of the hypothalamic–pituitary–thyroid axis and can be used to simulate the dynamic behavior of thyroidal hormone concentrations under different (euthyroid and non-euthyroid) conditions, and sometimes also for clinical decision-making (7). Furthermore, in Ref. (8), a method is proposed to compute personalized euthyroid setpoints that can be used for individualized diagnosis and treatment of thyroid diseases. While this static model is appealing due to its simplicity (only two parameter values have to be estimated), it does not consider any dynamic phenomena in the HPT axis, which are, however, of great importance for a deepened understanding of the HPT axis and ultimately the development of personalized optimal medication strategies. Another drawback is the absence of any consideration of *T*_3_, which has been shown to be significant not only as a key actor in the hypothalamic–pituitary–thyroid feedback loop (4, 9) but also in maintaining a good quality of life (5).

The main objective of this paper is an improved mathematical modeling of the HPT axis in order to obtain a more detailed understanding of the dynamic phenomena occurring in thyroid hormone homeostasis. In particular, as a first contribution, we extend the model originally developed in Ref. (1, 2) in order to incorporate new insights obtained through several recent clinical studies. In particular, we incorporate a direct *TSH*-*T*_3_ path inside the thyroid, accounting for the central *T*_3_ production by the thyroid. Existence of such a *TSH*-*T*_3_-shunt was hypothesized and demonstrated in several experiments and clinical observations (10–15). In Ref. (10), it was shown that *L*-*T*_4_-treated athyreotic patients exhibit decreased *FT*_3_ concentrations despite normal free thyroxine (*FT*_4_) levels, which would not be the case if peripheral *FT*_3_ was mainly produced by deiodination of peripheral *FT*_4_. Furthermore, the sum activity of step-up deiodinases (*G_D_*) is positively correlated with the *TSH* concentration (11) and with the thyroidal volume (12) and significantly decreases after thyroidectomy. These observations suggest that besides the peripheral *T*_4_/*T*_3_ conversion, also *TSH*-stimulated deiodinases inside the thyroid contribute to the total *T*_3_ production. In our work, we show that the extended model including such a *TSH*-*T*_3_-shunt is in good accordance with various clinical observations. For example, we show that the *FT*_3_ concentration shows a clear circadian pattern, as was observed *in vivo* in Ref. (16). Notably, this is not the case in the previous model, which did not include the *TSH*-*T*_3_-shunt.

As a second main contribution of this paper, we perform a sensitivity analysis of the derived model. Loosely speaking, the (first-order) sensitivities are a measure for how “sensitive” certain system states (i.e., *TSH* or hormone concentrations) are with respect to changes in certain parameters (such as, e.g., the thyroid’s secretory capacity *G_T_*). These sensitivities reveal structural insight into the functionality of the hypothalamic–pituitary–thyroid axis and can provide explanations for certain clinical observations. For example, we show that the sensitivity of *TSH* with respect to *G_T_* is much higher for low values of *G_T_* (i.e., in hypothyroidism) than for high values of *G_T_* (i.e., in hyperthyroidism). This fact can be used to explain why in clinical practice, *TSH* concentrations may significantly vary beyond the upper limit of the reference range despite normal thyroid function.

The remainder of this paper is structured as follows. Section 2 presents the extended mathematical model and discusses the identification of the required (additional) parameters. In Section 3, we show simulation results of the derived model and discuss the observed properties (such as the existence of a circadian rhythm in *FT*_3_ concentrations). A sensitivity analysis of *TSH*, *FT*_4_, and *FT*_3_ concentrations with respect to different parameters is performed in Section 4. Finally, we conclude the paper in Section 5.

## 2 Presentation of the Extended Model and Parameter Identification

**Figure 1:**
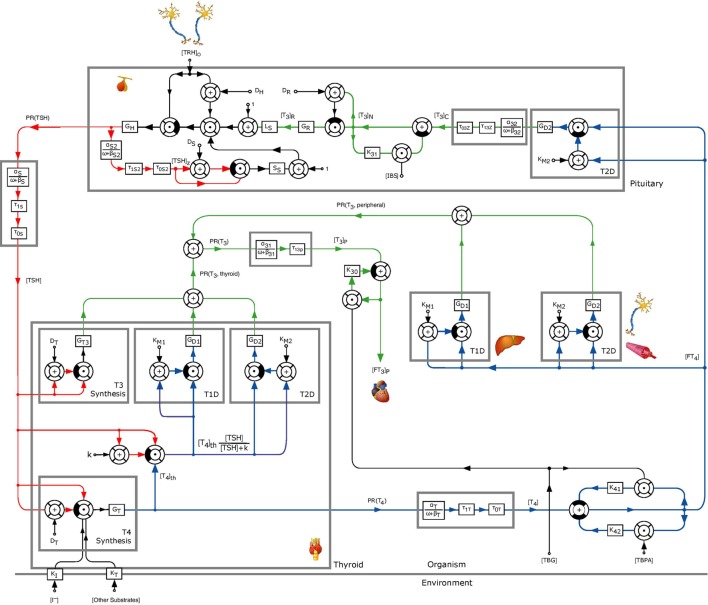
Block diagram of the thyrotropic feedback control loop with an additional *TSH*-*T*_3_-shunt, adapted from Ref. (1, 2). Except for *G_T_*_3_, *k*, and *G_D_*_1_, all parameters were adopted from the model in Ref. (1, 2). The parameters *G_D_*_1_ and *G_T_*_3_ were estimated to obtain an optimal (in a least squares sense) fit to measured *in vivo FT*_3_-concentrations. To this end, the value of *k* was normalized to 1 mU/l.

As outlined above, several clinical observations have led to the hypothesis that a direct, *TSH*-stimulated path exists for *T*_3_ production inside the thyroid, which we now incorporate into the mathematical model from Ref. (1, 2). The extended model including this *TSH*-*T*_3_-shunt is illustrated in Figure 1, see Section S1 in the Supplementary Material for a mathematical description of the underlying differential equations. To this end, both intrathyroidal conversion of *T*_4_ into *T*_3_ via type 1 and 2 5′-deiodinases as well as a direct synthesis of *T*_3_ are modeled (see upper three blocks in the “Thyroid” block in Figure 1). Both mechanisms are stimulated by *TSH* and modeled via nonlinear Michaelis–Menten–Hill kinetics, see Section S1 in the Supplementary Material for further details.

Most of the parameters of the extended model can be taken from the model in Ref. (1, 2), where the parameters have been estimated according to known physical quantities (such as the half-life period of certain substances, etc.) or have been identified using data measured *in vivo*. A detailed listing of these parameters can be found in the Tables S1–S3 in Supplementary Material. Some of the parameters were calibrated according to average population data, and hence the resulting model can be interpreted to be a functional model of some generic euthyroid subject. Clearly, personalized model identification would be highly valuable for individualized clinical decision-making and the development of personalized optimal medication strategies. For this, however, sufficient data such as individual dynamic trajectories of hormone concentrations would be needed to avoid overfitting. We note that, while the present report deals mainly with average population data, the observed phenomena are in good accordance with individual samples (9).

For the extended model, the new parameters *G_T_*_3_ and *k* have to be determined. Also, the sum activity of the type 1 5′-deiodinase, *G_D_*_1_, has to be re-estimated. This is the case since the extended model considers the additional *T*_3_ secretion inside the thyroid, while in the original model, *G_D_*_1_ was calibrated by only considering peripheral *T*_3_ production, and hence *G_D_*_1_ had been estimated too high. In order to obtain the parameters *G_T_*_3_ and *G_D_*_1_, a least squares estimation was performed, fitting the *FT*_3_-output of the presented model to *FT*_3_ measurements of a clinical study. Although there is no unique solution in case that only single *FT*_3_ measurements are available, it provides a set of optimal parameters, which could be further reduced to a unique solution if additional measurements were available (compare the detailed discussion below). In order to perform the least squares estimation, the equilibrium *FT*_3_ level predicted by the extended model, in the following denoted by *FT*_3,*eq*_, can be computed in dependence of the parameters by solving a cubic polynomial (see Section S1 in the Supplementary Material for a more detailed description). For this computation, we set *TRH* to a constant value (later, for the dynamic analysis *TRH* is varying in a sinusoidal fashion). This equilibrium value is then fitted in a least-squares sense to real measurement data resulting from 1,121 untreated patients of the clinical study in Ref. (11). In particular, this is achieved by minimizing the following cost function with respect to the parameters *G_T_*_3_, *k*, and *G_D_*_1_:

(1) $$J\left(G_{T3},k,G_{D1}\right)=\sum_{i=1}^{1121}\;{\left({\mathrm{FT}}_{3,i}-{\mathrm{FT}}_{3,\mathrm{eq}}\left(G_{T3},k,G_{D1}\right)\right)}^{2}$$

**Figure 2:**
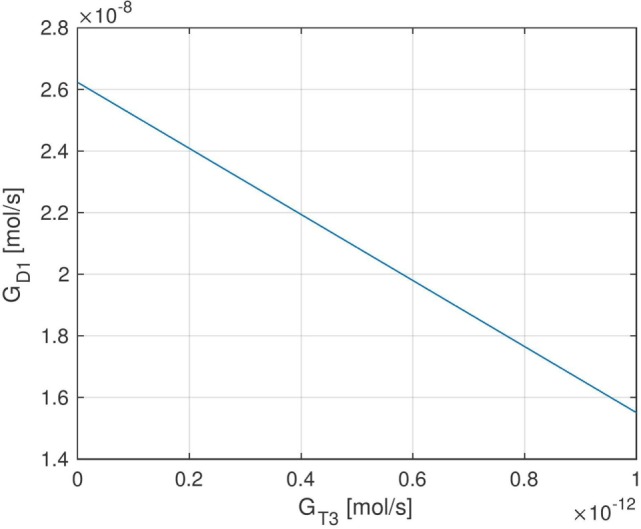
Set of optimal (in a least-squares sense) parameters *G_T_*_3_ and *G_D_*_1_ when normalizing the parameter *k* to 1 mU/l. Due to the affine dependence of *FT*_3,*eq*_ (G*_T_*_3_, *k*, *G_D_*_1_) on *G_T_*_3_ and *G_D_*_1_, the set of optimal parameters is contained in a one-dimensional affine subspace of $R^{2}$.

Here, *FT*_3,*i*_ denotes the measured *FT*_3_-concentration of the i-th patient, and *FT*_3,*eq*_ (*G_T_*_3_, *k*, *G_D_*_1_) is the equilibrium *FT*_3_-level predicted by the model depending on the parameters *G_T_*_3_, *k*, and *G_D_*_1_. The other parameters that are needed to compute *FT*_3,*eq*_ are adopted from Ref. (1) (see Tables S1–S3 in the Supplementary Material). The optimal solution to the above optimization problem can be determined analytically and is given by $FT_{3,\mathrm{eq}}\left(G_{T3},k,G_{D1}\right)\;=\;\bar{{\mathrm{FT}}_{3}}$, where $\bar{{\mathrm{FT}}_{3}}$ is the mean value of the 1,121 *FT*_3_ measurements. Using the derived formula for *FT*_3,*eq*_(*G_T_*_3_, *k*, *G_D_*_1_) (see Section S1 in the Supplementary Material), this results in different (infinitely many) optimal parameter combinations for *G_T_*_3_, *k*, and *G_D_*_1_. For example, normalizing *k* to $1\frac{\mathrm{mU}}{l}$ (which will be used in the following), the optimal parameter combinations for *G_T_*_3_ and *G_D_*_1_ can be seen in Figure 2.

Different (optimal) parameter combinations for *G_T_*_3_ and *G_D_*_1_ result in different fractions of thyroidal and peripheral *T*_3_ production. For example, $G_{D1}=22\frac{\mathrm{nmol}}{s}$ and $G_{T3}=394\frac{\mathrm{fmol}}{s}$ approximately lead to 80% *T*_3_ production from peripheral conversion of *FT*_4_ and approximately 20% *T*_3_ production from intrathyroidal secretion, corresponding to the values suggested by Ref. (17, 18). On the other hand, also, a higher or lower fraction of intrathyroidal *T*_3_ production is possible, depending on the values of *G_T_*_3_ and *G_D_*_1_. In particular, higher values of *G_T_*_3_ and lower values for *G_D_*_1_ result in a higher fraction of intrathyroidal *T*_3_ production and vice versa. For the dynamic simulation of the model and the sensitivity analysis in the following sections, we (mostly) use the values $G_{D1}=22\frac{\mathrm{nmol}}{s}$ and $G_{T3}=394\frac{\mathrm{fmol}}{s}$, and we comment when certain results qualitatively change if other parameter values for *G_D_*_1_ and *G_T_*_3_ are used.

The above discussed non-uniqueness in the optimal parameter fit is due to the fact that the model is not fully identifiable given the measured data. Namely, *FT*_3_ is the only hormone that is affected by the parameters *G_T_*_3_, *k*, and *G_D_*_1_, and we only have stationary measurements available. Furthermore, in the above estimation, we made the simplifying assumption that peripheral and thyroidal deiodinase activities (*G_D_*_1_ and *G_D_*_2_) are the same, which might in general not be the case. Identifying the corresponding parameters separately would result in a possibly better parameterized model, which is, however, again not possible given only the stationary *FT*_3_ measurements. On the other hand, if we had additional data such as dynamic hormone concentration trajectories or additional measurements (e.g., intrathyroidal hormone concentrations), the above described non-uniqueness in the parameter estimation could be removed and also different parameter values for thyroidal and peripheral deiodinase activity could be identified, allowing for a more exact parameterization of the model. This would be an interesting topic for future research, however, such *in vivo* data are difficult to obtain and are typically not available. Moreover, the presented model does not consider membrane transport processes between thyroidal and peripheral tissue. Incorporating such processes by means of a compartment model would further increase the quality of our model, yet, this would yield additional parameters, which had to be identified. Nevertheless, as we will show in the following sections, the extended model with the parameters as identified in this section is a clear improvement compared to the previous model, allowing for a better reproduction and interpretation of various clinically observed phenomena.

## 3 Dynamic Properties of the Extended Model

In the following, we simulate the extended model and analyze and interpret the obtained results. First, some simulation runs are carried out to illustrate the role of the *TSH*-*T*_3_-shunt in obtaining a circadian rhythm in the *FT*_3_-concentration. Afterwards, we investigate the delay between *TSH* and *FT*_3_, which has been observed in several clinical studies [e.g., Ref. (16, 19)].

### 3.1 Dynamic Simulation

As detailed in the previous section, the intrathyroidal *T*_3_ secretion is composed of two mechanisms, namely intrathyroidal conversion of *T*_4_ into *T*_3_ via type 1 and 2 5′-deiodinases (upper middle and right block inside the thyroid in Figure 1) as well as a direct synthesis of *T*_3_ (upper left block inside the thyroid in Figure 1). In the dynamic simulation using the parameters as identified in Section 2, the intrathyroidal contribution to the total *T*_3_ secretion rate was composed as follows:

$$\begin{array}{ll} \frac{\mathrm{Output of block ”T3 Synthesis”}}{\mathrm{PR}\left(T_{3},\mathrm{thyroid}\right)} & =79.7\%\\ \frac{\mathrm{Output of block ”T1D”}}{\mathrm{PR}\left(T_{3},\mathrm{thyroid}\right)} & =20.3\%\\ \frac{\mathrm{Output of block ”T2D”}}{\mathrm{PR}\left(T_{3},\mathrm{thyroid}\right)} & =0.002\% \end{array}$$

Hence, with the parameters identified in Section 2, the main thyroidal source to *T*_3_-production is direct *T*_3_-synthesis via Michaelis–Menten–Hill kinetics, represented by the block “T3 Synthesis” in Figure 1. On the other hand, deiodination by type 2 5′-deiodinases has a negligible effect only, since the sum activity of type 2 5′-deiodinases is much smaller compared to that of type 1 5′-deiodinases. In case that a different optimal combination of parameters *G_T_*_3_ and *G_D_*_1_ is used (compare Section 2), the above results change accordingly, i.e., a higher value of *G_D_*_1_ yields a higher contribution of the deiodination by type 1 5′-deiodinases to the thyroidal *T_3_*-production. However, this also causes a change in the ratio between thyroidal and peripheral *T_3_* production, as discussed in Section 2.

**Figure 3:**
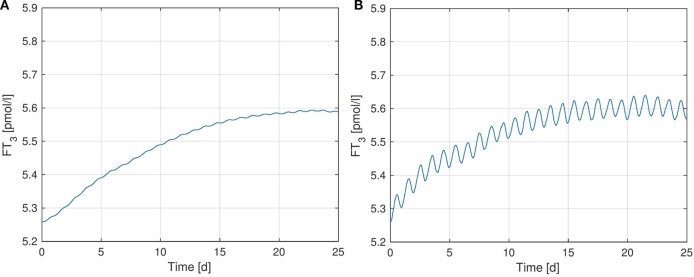
*FT*_3_-plots $[\frac{\mathrm{pmol}}{l}]$ over a simulation horizon of 25 days for several configurations of the *TSH*-*T*_3_-Shunt. The parameters *G_T_*_3_ and *G_D_*_1_ are identified via least squares optimization, separately for each model configuration. **(A)** No shunt included. **(B)** Full *TSH*-*T*_3_-shunt.

Figure 3 shows simulated *FT_3_*-plots, where we further investigated the effect of the *TSH*-*T_3_*-shunt on the dynamic behavior of *FT_3_*.^1^ In particular, Figure 3A shows simulation results using the previous model from Ref. (1) without the *TSH*-*T_3_*-shunt whereas in Figure 3B, the full *TSH*-*T_3_*-shunt as described in the previous section is included. For each of the two scenarios (i.e., for the corresponding models), we separately identified the (in a least-squares-sense) optimal parameter(s): *G_D_*_1_ for the model corresponding to Figure 3A and *G_D_*_1_ as well as *G_T_*_3_ for the model corresponding to Figure 3B. The exact values of these parameters for the different model configurations can be seen in Table S4 in Supplementary Material.

We observe that the *TSH*-*T*_3_-shunt causes a clear circadian oscillation in the *FT*_3_ concentration, which is not (or only very weakly) present without considering intrathyroidal *T*_3_ secretion. Such a circadian rhythm in *FT*_3_ concentration has been observed *in vivo* in several clinical studies [see, e.g., Ref. (16, 19)], and hence our simulation results again support existence of the *TSH*-*T*_3_-shunt.

Quantitatively, the oscillation amplitude of the measured *in vivo FT*_3_ concentration in Ref. (16) is approximately six times as big as the amplitude observed in the simulated model (see Figure 3B). This difference might be due to the assumptions we made for the identification in Section 2 (same values for *G_D_*_1_, *G_D_*_2_, *K_M_*_1_, and *K_M_*_2_ inside the thyroid and the peripheral tissue). Namely, if thyroidal deiodination activity and/or *G_T_*_3_ were higher than computed in Section 2, without increasing the peripheral deiodination activity as well, we would obtain a larger oscillation amplitude in *FT*_3_ concentration. Nevertheless, the fact that a clear circadian pattern arises in the simulations when including the *TSH*-*T*_3_-shunt into the model is a clear indicator supporting both its existence as well as the fact that thyroidal *T*_3_ secretion is stimulated by *TSH*.

### 3.2 Delay of *FT_3_* w.r.t. *TSH*

The authors in Ref. (16) make the observation that *in vivo FT*_3_-measurements follow a clear circadian pattern, which is approximately 90 min delayed w.r.t. *TSH*; this number can also vary between different individuals (19). As already mentioned in the previous section, the *FT*_3_-level obtained by the model in Figure 1 including intrathyroidal *T*_3_-secretion shows a clear circadian pattern. In this section, we investigate how the delay between *TSH* and *FT*_3_ in the presented model is influenced by this newly incorporated mechanism.

A dynamic simulation with the same setup as in Section 3.1 yields the following: when incorporating the *TSH*-*T*_3_-shunt into the model, *FT*_3_ is delayed w.r.t. *TSH* by approximately 6 h, whereas the delay amounts to 13 h in the previous model, which did not incorporate this mechanism. These observed values can be explained as follows. The phase shift between *FT*_3_ and *TSH* in our model mainly results from the first order lag elements $\frac{\alpha }{i\omega +\beta }$ modeling peripheral *T*_3_ and *T*_4_ secretion (i.e., the ones with parameters *α*_31_, *β*_31_, and *α_T_*, *β_T_*, respectively in Figure 1). The phase shift of the output signal of such a first order lag element for a given sinusoidal input signal with frequency *ω* depends on the parameter *β* and is given as follows:

(2) $$\mathrm{phase}=\arctan\left(-\frac{\omega }{\beta }\right).$$

In our case, $\omega =\frac{2\pi }{T}$ where *T* = 86,400 *s* is the circadian period of 1 day. The delay between the output and input signal is now computed by simply relating the phase shift to the period length $T:\mathrm{delay}=-\mathrm{phase}\cdot \frac{T}{2\pi }$. For the given parameter values *α*_31_, *β*_31_, and *α_T_*, *β_T_* of *T*_3_- and *T*_4_-generation, respectively, we obtain a delay of approximately 5.5 and 6 h, respectively.

The above observed delay of *FT*_3_ w.r.t. *TSH* can now be explained as follows. In the previous model not including the *TSH*-*T*_3_-shunt, the circadian oscillation has to pass through both first order lag elements for peripheral *T*_4_ and *T*_3_ production, resulting in a high delay w.r.t. *TSH*. On the other hand, the fraction of *T*_3_ secreted inside the thyroid does not exhibit the delay caused by peripheral *T*_4_ production and hence exhibits a much shorter delay w.r.t. *TSH*. Interestingly, the observed delay of total *T*_3_ (approximately 6 h) mainly seems to be determined by the shorter one resulting from intrathyroidal *T*_3_ production, although approximately 80% of the total *T*_3_-production results from peripheral *FT*_4_-deiodination and only 20% from intrathyroidal secretion. The reason for this is that as explained above, the circadian rhythm of *FT*_3_ is mainly induced by intrathyroidal *T*_3_ secretion. Namely, the ratio of the amplitude and the mean value equals 0.3% for the peripheral *T*_3_ production rate *PR*(*T*_3_, *peripheral*) and 23% for the thyroidal *T*_3_ production rate *PR*(*T*_3_, *thyroid*). Thus, the phase of *FT*_3_ is almost solely characterized by the phase of thyroidal *T*_3_-production, and hence the delay of *FT*_3_ w.r.t. *TSH* is determined by the phase shift of only one first order lag element when the shunt is included, compared to two without the shunt. To conclude, the inclusion of the *TSH*-*T*_3_-shunt into the model significantly reduces the delay of *FT*_3_ w.r.t. *TSH*. While the absolute numbers are still too high compared to the observed *in vivo* delays (16, 19), this is again a clear indicator for the existence of the *TSH*-*T*_3_-shunt.

## 4 Sensitivity Analysis and Stationary Dependencies

In this section, we perform a sensitivity analysis of the previously presented mathematical model of the hypothalamic–pituitary–thyroid feedback loop (see Figure 1). Sensitivity analysis is a tool for determining how a certain parameter influences the trajectories resulting from simulation of the model, i.e., from the solution of the underlying system of differential equations, and in particular, how “sensitive” these trajectories are with respect to certain parameter changes. In the following, we give a brief non-formal introduction to sensitivity analysis and refer to the Section S2 in Supplementary Material for a more complete and formal description.

To define sensitivities, consider the following vector-valued ordinary differential equation with parameter vector *p*:

(3) $$\dot{x}=f\left(t,x,p\right),\;x\left(t_{0}\right)=x_{0}.$$

The first-order sensitivity function^2^ is now defined as $S\left(t\right)={\left.\frac{\partial x\left(t,p\right)}{\partial p}\right|}_{p=p_{0}}$, where *p*_0_ is some nominal (constant) parameter value. The sensitivity function *S*(*t*) is a time-dependent matrix with as many rows as the dimension of *x* and as many columns as the dimension of *p*. Under some assumptions (smoothness, existence of solutions, …), it can be shown that *S* satisfies the following differential equation, which is solved simultaneously with the state equation (3), see Ref. (20).

(4) $$\begin{array}{l} \dot{x}=f\left(t,x,p_{0}\right)\\ \dot{S}={\left[\frac{\partial f\left(t,x,p\right)}{\partial x}\right]}_{p=p_{0}}\cdot S+{\left[\frac{\partial f\left(t,x,p\right)}{\partial p}\right]}_{p=p_{0}}\\ x\left(t_{0}\right)=x_{0},\;S\left(t_{0}\right)=0. \end{array}$$

The initial sensitivity, i.e., *S*(*t*_0_), is set to zero since the states’ initial values are independent of the parameters. The above presented mathematical model of the hypothalamic–pituitary–thyroid feedback loop (see Figure 1) includes 36 parameters. With 5 states (pituitary *TSH* and *T*_3_ as well as peripheral *TSH*, *T*_4_, and *T*_3_ concentrations), this makes a total of 180 different sensitivity curves - for one specific nominal parameter configuration *p*_0_. In the following, we only analyze a few interesting curves to obtain some new insights. Of course, if desired, one could analogously analyze further sensitivities of other state and parameter pairs. In order to be able to employ the standard sensitivity analysis tools described above, the time delays in the hypothalamic–pituitary–thyroid (HPT) axis model are neglected.

### 4.1 Sensitivity of *T_4_* w.r.t. *G_T_*

**Figure 4:**
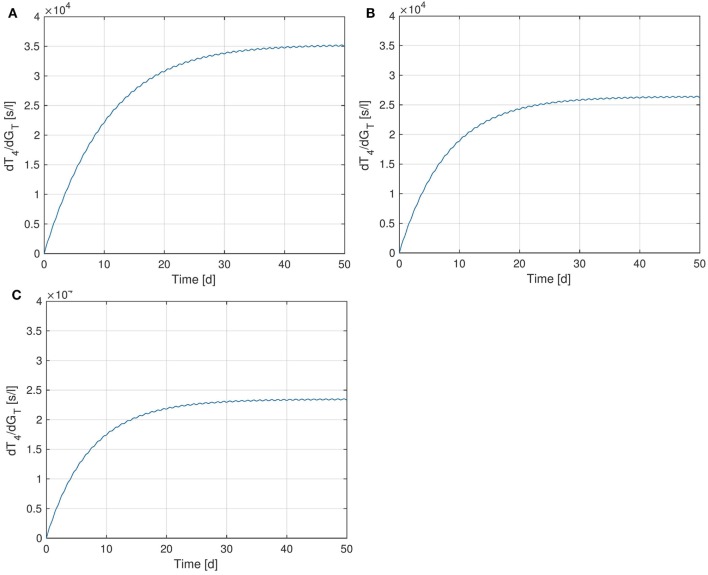
Sensitivity of *T*_4_ w.r.t. *G_T_* for different values of *G_T_*. **(A)** $G_{T}=1.2\cdot 10^{-12}\frac{\mathrm{mol}}{s}$, **(B)** $G_{T}=3.375\cdot 10^{-12}\frac{\mathrm{mol}}{s}$ - nominal value, **(C)** $G_{T}=5\cdot 10^{-12}\frac{\mathrm{mol}}{s}$.

**Figure 5:**
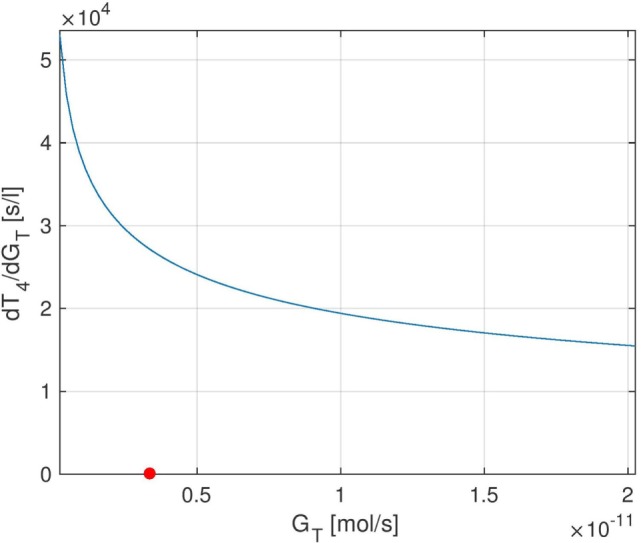
Stationary sensitivity of *T*_4_ w.r.t. *G_T_* as a *G_T_*-dependent function. The red point indicates the nominal *G_T_*-value from Ref. (1).

We start by examining the sensitivity of peripheral *T*_4_ with respect to the thyroid’s secretory capacity *G_T_*. In Figure 4, several plots of the sensitivity $\frac{\partial T_{4}}{\partial G_{T}}\left(t\right)$ are shown with different *G_T_*-values, corresponding to different parameter values *p*_0_ in equation (4).^3^ Note that the sensitivity curves do not exhibit large variations over the day, i.e., only show a small circadian oscillation. It can be seen that different values of *G_T_* result in different sensitivities of *T*_4_ with respect to *G_T_*. For example, a low value of *G_T_* (which can be seen as a simple modeling of hypothyroidism) causes an increase of the sensitivity, whereas a high *G_T_* value (which can be seen as a simple modeling of hyperthyroidism) causes a decrease of the sensitivity. This means that larger fluctuations in *T*_4_ can be expected at the lower end of its euthyroid reference range (compare Section 4.3). This observation can compactly be expressed for a wide range of *G_T_*-values by investigating the stationary sensitivity (i.e., $\underset{t\to \infty }{\lim}\;\frac{\partial T_{4}}{\partial G_{T}}\left(t\right)$). Figure 5 shows it as a function of the parameter *G_T_*. A comparison between Figures 4 and 5 shows that the stationary sensitivity, indeed, is the limit of the sensitivity curve for *t* → ∞.

### 4.2 Sensitivity of *TSH* w.r.t. *TRH*

**Figure 6:**
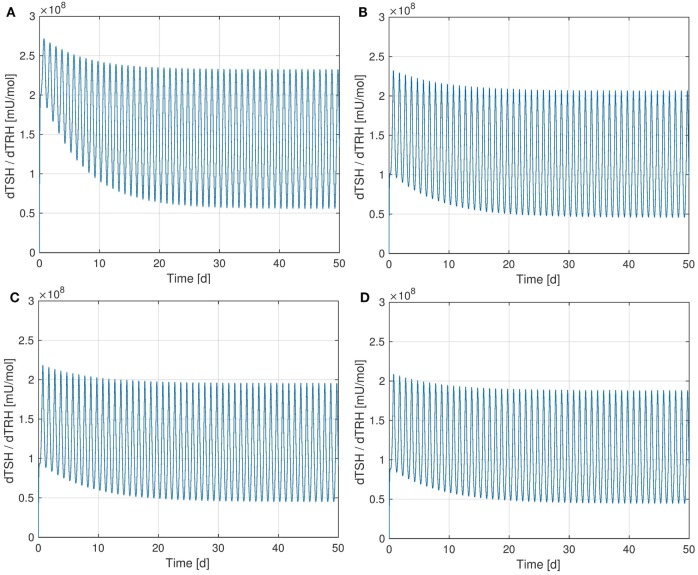
Sensitivity of *TSH* w.r.t. *TRH* for different values of *S_S_*. **(A)** $S_{S}=0\frac{l}{\mathrm{mU}}$, **(B)** $S_{S}=50\frac{l}{\mathrm{mU}}$, **(C)** $S_{S}=100\frac{l}{\mathrm{mU}}$ - nominal value, **(D)** $S_{S}=200\frac{l}{\mathrm{mU}}$.

It is interesting to observe that the Ultra-Short-Feedback loop (i.e., the lower left part inside the pituitary in Figure 1, compare Ref. (2)) has a significant influence on the sensitivity of *TSH* w.r.t. TRH. Figure 6 shows the curves of this sensitivity for different values of *S_s_*. It can be seen that an increase in *S_s_* causes a decrease in the sensitivity.

In the considered HPT axis model, *TRH* is treated as a time-dependent input that comes from the hypothalamus. A disturbance in the system could lead to a change in the *TRH* concentration arriving at the pituitary. Apparently, the Ultra-Short-Feedback increases the robustness of the *TSH* production w.r.t. changes in portal *TRH*. If the additional feedback is absent (i.e., *S_s_* = 0), the sensitivity is significantly higher than in the nominal case $\left(S_{S}=100\frac{l}{\mathrm{mU}}\right)$.

### 4.3 Stationary Dependencies of *TSH* and *T_4_* on *G_T_*

In the following, the influence of the thyroid’s secretory capacity *G_T_* on the equilibrium concentrations of *TSH* and *T*_4_ is analyzed. To this end, we solve the system’s stationary equations (i.e., for *t* → ∞) for the different hormones and plot the resulting equilibrium hormone levels as functions of *G_T_*. The slopes of these functions are exactly the entries of the stationary sensitivity matrix lim_*t* → ∞_*S*(*t*). For example, the stationary sensitivity $\underset{t\to \infty }{\lim}\;\frac{\partial T_{4}}{\partial G_{T}}\left(t\right)$ for a given value of *G_T_* is equal to the derivative of the curve *T*_4_(*G_T_*) w.r.t. *G_T_*, which we treat in the following.

**Figure 7:**
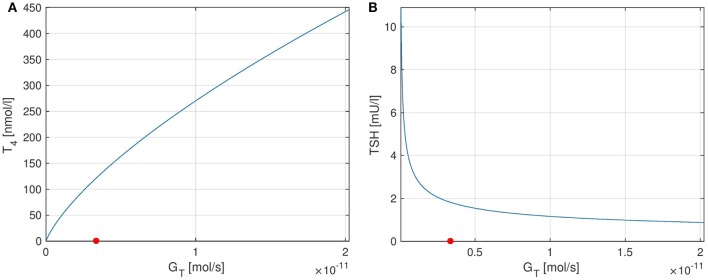
Plots of equilibrium *T*_4_ and *TSH* levels depending on the thyroid’s secretory capacity *G_T_*. The red point in the figures indicates the nominal *G_T_*-value from Ref. (1). **(A)** Equilibrium *T*_4_, **(B)** equilibrium *TSH*.

The curves of *T*_4_ and *TSH* depending on *G_T_* are shown in Figure 7. One can see that the parameter *G_T_* can be used as a measure of hypo- or hyperthyroidism (1). The equilibrium *T*_4_-concentration increases almost linearly with *G_T_*. Furthermore, we have high *TSH*-levels for low values of *G_T_*, i.e., in hypothyroidism, and vice versa. This is a well-known fact, which is usually used in clinical decision-making for the determination of subclinical thyroid diseases. Another interesting fact is that the magnitude of the sensitivity of *TSH* w.r.t. *G_T_* (which is the slope of Figure 7B) is high for low values of *G_T_* and vice versa. This means that *TSH* is much more sensitive to fluctuations in the thyroid’s secretory capacity if *G_T_* is low. This fact can be used to interpret the following clinical observation. In practice, *TSH* concentrations may be misleading, especially, if located slightly above the vague upper limit of the reference range. A reason for this could be that as discussed above, *TSH* is much more sensitive to fluctuations in the thyroid’s secretory capacity (e.g., due to different iodine supply and other influences) at the upper limit of its (euthyroid) reference range than at its lower limit.

### 4.4 Sensitivity of *FT_3_* w.r.t. *G_T_*

**Figure 8:**
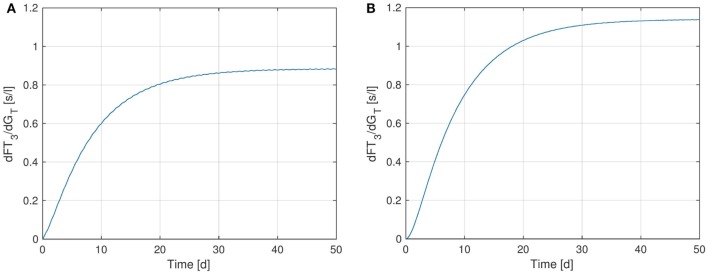
Sensitivity of *FT*_3_ w.r.t. *G_T_* for two versions of the HPT axis model: one incorporating the *TSH*-*T*_3_-shunt and one without this extension. **(A)** Full *TSH*-*T*_3_-shunt, **(B)** no shunt included.

Finally, we perform a sensitivity analysis for *FT*_3_ w.r.t. the parameter *G_T_*. Figure 8 shows the sensitivity curve, where in Figure 8A, the extended model including the *TSH*-*T*_3_-shunt was used, whereas Figure 8B uses the previous model without the shunt. It can be seen that the sensitivity of *FT*_3_ w.r.t. *G_T_* decreases when the shunt is included. This is to be expected since in the extended model, a direct synthesis of *T*_3_ (upper left block inside the thyroid in Figure 1) is included, which is independent of *G_T_*, i.e., from the thyroid’s secretory capacity for *T*_4_. Another interesting fact is that we can observe a small circadian rhythm in Figure 8A whereas the plot (Figure 8B) seems not to be affected by this. This confirms the observations we made in Section 3.1, namely that incorporating intrathyroidal *T*_3_-secretion causes a circadian rhythm in *FT*_3_ and hence also in the sensitivity w.r.t. *G_T_*.

**Figure 9:**
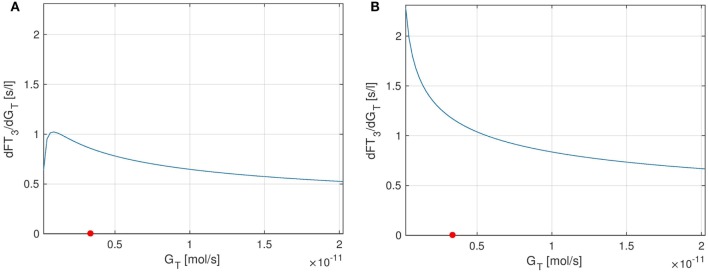
Plots of the stationary sensitivity of *FT*_3_ w.r.t. the parameter *G_T_* as a function of the thyroid’s secretory capacity *G_T_*. Two configurations of the model are shown: one including the *TSH*-*T*_3_-shunt and one without the shunt. The red point in the Figures indicates the nominal *G_T_*-value from Ref. (1). **(A)** Full *TSH*-*T*_3_-shunt, **(B)** no shunt included.

As for *T*_4_ and *G_T_*, we can also analyze the stationary sensitivity $\underset{t\to \infty }{\lim}\;\frac{\partial FT_{3}}{\partial G_{T}}\left(t\right)$ for different values of *G_T_*. In Ref. (10), the outcomes of a clinical study lead to the observation that the dependency of *T*_3_-generation on *G_T_* is lower than that predicted by a model, which does not include a *TSH*-*T*_3_-shunt. Indeed, comparing Figures 9A,B, one can see that the sensitivity of *FT*_3_ w.r.t. *G_T_* significantly decreases when the shunt is incorporated into the model, i.e., *T*_3_ production is less sensitive to fluctuations in the thyroid’s secretory capacity if the shunt is included. As already mentioned above, this seems plausible since we now have a completely *G_T_*-independent path from *TSH* to *FT*_3_ (upper left block inside the thyroid in Figure 1).

## 5 Conclusion and Outlook

In this work, a mathematical model of the hypothalamic–pituitary–thyroid feedback loop was extended to include *TSH*-stimulated intrathyroidal *T*_3_-secretion. The hypothesis of the existence of such a *TSH*-*T*_3_-shunt has been brought forward in various recent clinical studies. Our results show that the hypothesized mechanism can indeed explain various clinical findings. In particular, we have shown that intrathyroidal *T*_3_-secretion results in a clear circadian pattern of peripheral *FT*_3_, which has been observed *in vivo* in, e.g., Ref. (16, 19), and which is not the case without the incorporation of such a *TSH*-*T*_3_-shunt. Also, a sensitivity analysis revealed that the sensitivity of peripheral *FT*_3_ with respect to the thyroid’s secretory capacity for *T*_4_ is indeed lower when including intrathyroidal *T*_3_-secretion into the model, in accordance with the clinical study of Ref. (10).

While the present report deals primarily with technical aspects of the thyroid pituitary feedback regulation, a better understanding of the underlying control system is of high clinical interest and relevance. Currently, clinical diagnosis and treatment of thyroid disease heavily relies on an indirect approach assessing the pituitary *TSH* response rather than circulating free thyroid hormones, *FT*_3_ and *FT*_4_ (21). The application is based on the underlying assumption that pituitary *TSH* in equilibrium at all times provides an accurate mirror image of the peripheral hormones. However, recent evidence has challenged this simplistic tenet suggesting that the HPT axis is a much more dynamic system than has been previously thought (5, 22). In particular, the interrelationships between *FT*_3_, *FT*_4_, and *TSH* are less constantly fixed, rather conditional and contextualy adaptive (5, 22). Mathematical modeling presented in this study confirms and advances the theoretical framework that is emerging from recent clinical studies. Given the high prevalence of subclinical thyroid disorders in the population, being as high as 10% in middle aged women, the epidemiological and therapeutic implications are substantial. From the performed sensitivity analysis in the present study, important insights into the functionality of the HPT axis have been obtained. These include the robustification of *TSH* production through the ultrashort feedback loop in the pituitary, as well as a possible explanation why in clinical practice, diagnosis of wrong subclinical hypothyroidism is much more common than diagnosis of wrong subclinical hyperthyroidism.

In particular, the upper reference limit for *TSH* has been a matter of fierce debate for a decade (23). According to our models, the issue appears to be more fundamentally rooted. This relates to a substantial error rate, depending on the statistical analytical technique used, in the conventional disease classification based solely on statistical *TSH* abnormality (24). The relative variability in *TSH* rises even further with higher *TSH* concentrations in subclinical hypothyroidism (25). Recent guidelines have raised the clinical threshold for therapeutic intervention in subclinical hypothyroidism to a *TSH* level of 10 mU/l, whereas the laboratory-based disease definition continues to rely on the upper reference limit of approx. 4 mU/l (21). A better understanding and refined mathematical expression of hypothalamic–pituitary regulation in allostatic reactions (22), in thyrotropic insufficiency (26), and in situations of imminent thyroid failure (9, 27) as well as in their interactions may help reconcile this discrepancy that poses a considerable challenge to clinical decision-making.

Future work should focus on the further extension of the model to include currently unmodeled phenomena and mechanisms, such as, e.g., non-classical thyroid hormone signaling (28) and compartment models for the incorporation of membrane transport processes, which are increasingly understood as a regulatory element in their own right (29, 30). We would also aim to define the steady-state more narrowly and precisely for individual subjects under different conditions in an attempt to reduce the high uncertainty surrounding *TSH* measurements at the upper limit of its reference range. In general, obtaining further insight into the overall functionality of the hypothalamic–pituitary–thyroid feedback loop and developing suitable and detailed enough mathematical models might eventually pave the way for designing optimal medication strategies for various non-euthyroid states of human hormone homeostasis.

## Author Contributions

JB and MM drafted the manuscript. JB performed the simulations using Matlab/Simulink. Figure 1 was designed by JD and modified by JB, all other figures were created by JB. The deidentified data used for the parameter identification was provided by RH. All authors read and approved the manuscript.

## Conflict of Interest Statement

JD received funding and personal fees by Sanofi-Henning, Hexal AG, Bristol-Myers Squibb, and Pfizer and is co-owner of the intellectual property rights for the patent “System and Method for Deriving Parameters for Homeostatic Feedback Control of an Individual” (Singapore Institute for Clinical Sciences, Biomedical Sciences Institutes, Application Number 201208940-5, WIPO number WO/2014/088516). All other authors declare that the research was conducted in the absence of any commercial or financial relationships that could be construed as a potential conflict of interest.

## Footnotes

^1^The initial hormone values for the simulations shown in Figure 3 (and for all subsequent simulation runs) were chosen as the stationary mean values of the model, i.e. as the hormone values the model yields for a constant *TRH* input. Note, however, that the choice of initial values is not particularly important for the simulation, as long as they lie somewhere in the euthyroid range.

^2^The definition of *S* is such that it measures the sensitivity locally around a given nominal parameter value *p*_0_ along a solution trajectory of system (3), which is why it is typically called *first-order* sensitivity. In the following, for brevity, we just use the term sensitivity.

^3^For the simulation runs shown in Figure 4 and in all subsequent dynamic sensitivity curves, the initial sensitivity is set to zero [cf. also the initial condition *S*(*t*_0_)=0 in (4)]
