# Supplementary material for: Mathematical Modeling of the Pituitary–Thyroid Feedback Loop: Role of a TSH-T3-Shunt and Sensitivity Analysis
Source: Front Endocrinol (Lausanne). 2018 Mar 21;9:91. doi: 10.3389/fendo.2018.00091 (PMC5871688; doi:10.3389/fendo.2018.00091)
Supplement: Supplementary file 1 [file Presentation_1.PDF]

Supplementary Material: Mathematical modeling of the pituitary-thyroid feedback loop: role of a TSH-T_3-shunt and sensitivity analysis

Julian Berberich, Johannes W. Dietrich, Rudolf Hoermann and Matthias A. Müller *

*Correspondence:
Matthias A. Müller
matthias.mueller@ist.uni-stuttgart.de

S1 DIFFERENTIAL EQUATIONS OF THE EXTENDED MODEL

In the following, the differential equations of the extended model presented in Section 2 of the main article are described. Since the equilibrium values of this system are of particular interest for the parameter estimation of G_{D1}, k, and G_{T3}, we also show how to compute the equilibria depending on the parameters. The model can be expressed via the following system of five coupled nonlinear delay differential equations:

$$\frac{dT_4}{dt}(t) = \alpha_T \cdot G_T \cdot \frac{TSH(t-\tau_{0T})}{TSH(t-\tau_{0T}) + D_T} - \beta_T \cdot T_4(t) \tag{S1}$$

$$\begin{aligned}\frac{dT_{3P}}{dt}(t) = \alpha_{31} \Bigg(& G_{D1} \cdot \frac{FT_4(t)}{FT_4(t) + K_{M1}} + G_{D2} \cdot \frac{FT_4(t)}{FT_4(t) + K_{M2}} \\ & + G_{T3} \cdot \frac{TSH(t)}{TSH(t) + D_T} + G_{D1} \frac{T_{4,th}(t) \frac{TSH(t)}{TSH(t)+k}}{K_{M1} + T_{4,th}(t) \frac{TSH(t)}{TSH(t)+k}} \\ & + G_{D2} \frac{T_{4,th}(t) \frac{TSH(t)}{TSH(t)+k}}{K_{M2} + T_{4,th}(t) \frac{TSH(t)}{TSH(t)+k}} \Bigg) - \beta_{31} \cdot T_{3P}(t)\end{aligned} \tag{S2}$$

$$\frac{dT_{3c}}{dt}(t) = \alpha_{32} G_{D2} \cdot \frac{FT_4(t-\tau_{03Z})}{FT_4(t-\tau_{03Z}) + K_{M2}} - \beta_{32} \cdot T_{3c}(t) \tag{S3}$$

$$\begin{aligned}\frac{dTSH}{dt}(t) = & \\ & \frac{\alpha_S G_H \cdot TRH(t-\tau_{0S})}{(TRH(t-\tau_{0S}) + D_H)\left(1 + S_S \cdot \frac{TSH_z(t-\tau_{0S})}{TSH_z(t-\tau_{0S}) + D_S}\right)(1 + L_S \cdot T_{3R}(t-\tau_{0S}))} \\ & - \beta_S \cdot TSH(t)\end{aligned} \tag{S4}$$

$$\frac{dTSH_z}{dt}(t) = \frac{\alpha_{S2} G_H \cdot TRH\,(t-\tau_{0S2})}{\left(TRH\,(t-\tau_{0S2}) + D_H\right)\left(1 + S_S \cdot \frac{TSH_z(t-\tau_{0S2})}{TSH_z(t-\tau_{0S2})+D_S}\right)\left(1 + L_S \cdot T_{3R}\,(t-\tau_{0S2})\right)} - \beta_{S2} \cdot TSH_z\,(t) \tag{S5}$$

where

$$\begin{aligned} T_{4,th} &= G_T \cdot \frac{TSH}{TSH + D_T} \cdot \frac{s}{l} \\ FT_3 &= T_{3P} \cdot \frac{1}{1 + K_{30} \cdot TBG} \\ FT_4 &= T_4 \cdot \frac{1}{1 + K_{41} \cdot TBG + K_{42} \cdot TBPA} \\ T_{3N} &= T_{3c} \cdot \frac{1}{1 + K_{31} \cdot IBS} \\ T_{3R} &= G_R \cdot \frac{T_{3N}}{T_{3N} + D_R} \end{aligned} \tag{S6}$$

As described in Section 2 in the main text, the novelty of the presented model is the inclusion of intrathyroidal T_3 production, depicted by the three blocks "T1D", "T3 Synthesis" and "T2D" in Figure 1 in the article. This results in the last three terms inside the bracket on the right hand side of Equation (S2). Concerning the TSH-stimulated deiodination (corresponding to the last two terms inside the bracket on the right hand side of Equation (S2)), the following comments are in order. First, the thyroidal T_4 concentration, $T_{4,th}$, is modeled as a zero-th order process, i.e., given by (S6). This is a valid approximation under the assumption that the thyroidal T_4 production is fast compared to peripheral T_4 distribution. Second, the stimulation of the deiodinases by TSH is modeled by the additional multiplicative term $TSH/(TSH + D_T)$. This can be derived as follows. The considered deiodination is an enzymatic reaction and can be modeled as many basic enzymatic reactions by

$$\mathrm{d} + \mathrm{T_{4,th}} \rightleftharpoons \mathrm{dT_{4,th}} \longrightarrow \mathrm{d} + \mathrm{T_3}, \tag{S7}$$

compare, e.g., [1]. Here, d is the (activated) deiodinase concentration and $dT_{4,th}$ is the concentration of an intermediate complex between the deiodinase and thyroidal T_4. Using the quasi-steady-state assumption[1] for the complex $dT_{4,th}$, such a reaction scheme can be approximated by the well-known Michaelis-Menten-Hill kinetics $G_{D1}T_{4,th}/(K_{M1} + T_{4,th})$ with appropriate constants G_{D1} and K_{M1}, see, e.g., [1]. The activation/stimulation of the thyroidal deiodinases by TSH is now modeled by a reaction of the form

$$\mathrm{d}' + \mathrm{TSH} \rightleftharpoons \mathrm{d},$$

[1] In case that this assumption does not hold, approximation by a Michaelis-Menten-Hill kinetics might not be valid and other modeling approaches might be required, compare, e.g., [2].

where $\mathrm{d}^{'}$ is the concentration of an inactive form of deiodinase. Combining this equation with (S7) and using again the quasi-steady-state assumption (both for deiodinase activation and the complex $dT_{4,th}$) results in the term appearing in (S2), i.e.,

$$G_{D1}\frac{T_{4,th}\frac{TSH}{TSH+k}}{K_{M1}+T_{4,th}\frac{TSH}{TSH+k}}$$

for some constant $k > 0$.

We note that an interesting topic for future work would be to include a more precise model of the thyroid and thyroidal T_4 production, e.g., a compartmental model including membrane transporters, or modeling the dynamics of the second messenger $cAMP$.

The numerical values of the parameters for the model (S1)–(S5) are listed in Section S3. In order to obtain the equilibrium hormone levels of the system (S1) - (S5), we set all of the derivatives in equations (S1) - (S5) to zero and solve the resulting equations for the corresponding hormone values. Due to the complex structure and the many parameters of the system, this is rather cumbersome but straightforward, as outlined in the following.

After defining some intermediate parameters

$$a_1 = \frac{\alpha_T G_T}{\beta_T},\ \ a_3 = \frac{\alpha_{32} G_{D2}}{\beta_{32}},\ \ b_1 = \frac{1}{1 + K_{41}TBG + K_{42}TBPA}$$
$$c_2 = 1 + \frac{K_{M2}}{a_1 b_1},\ \ c_3 = D_T\frac{K_{M2}}{a_1 b_1}$$

and plugging T_4 from equation (S1) into (S3), one arrives at

$$T_{3c} = a_3\frac{TSH}{c_2 TSH + c_3}.$$

This can then be inserted into (S5) which, after performing some algebraic computations, leads to

$$TSH = \frac{g_1 TSH_z^2 + g_2 TSH_z + g_3}{g_4 TSH_z^2 + g_5 TSHz + g_6}, \tag{S8}$$

where we again define intermediate parameters as follows:

$$a_5 = \frac{\alpha_{S2} G_H}{\beta_{S2}},\ b_3 = \frac{1}{1 + K_{31} IBS},\ c_1 = \frac{TRH}{TRH + D_H},\ d_2 = L_S G_R b_3 a_3,$$
$$d_3 = b_3 a_3 + c_2 D_R,\ d_4 = c_3 D_R,\ d_5 = d_2 + d_3,\ d_6 = 1 + S_S,\ f_1 = c_1 a_5,$$
$$g_1 = -d_4 d_6,\ g_2 = f_1 d_4 - d_4 D_S,\ g_3 = f_1 d_4 D_S,\ g_4 = d_5 d_6,\ g_5 = -f_1 d_3 + d_5 D_S,$$
$$g_6 = -f_1 d_3 D_S,\ g_7 = \frac{\alpha_{S2} \beta_S}{\beta_{S2} \alpha_S}.$$

From equations (S4) and (S5), we can also conclude that $TSH_z = g_7 TSH$. Now, plugging this into (S8), we finally arrive at a cubic equation in TSH:

$$m_1 TSH^3 + m_2 TSH^2 + m_3 TSH + m_4 = 0 \tag{S9}$$

where

$$m_1 = g_4 g_7^2,\ m_2 = g_5 g_7 - g_1 g_7^2,\ m_3 = g_6 - g_2 g_7,\ m_4 = -g_3$$

Solving this cubic equation yields three (complex-valued) solutions for the equilibrium level of TSH. In practice, it turns out that for the most common parameter settings, there is, indeed, only one solution that is real-valued and positive and therefore physically reasonable.

Now, one can simply plug this solution for TSH into equations (S1), (S2), (S3), and (S5) to solve for the other steady-state values. Since the equilibrium value of free peripheral T_3 is of particular interest for the parameter identification, we state the formula for its computation, depending on the parameters G_{T3}, k, and G_{D1}.

$$\begin{aligned} FT_{3,eq}\left(G_{T3}, k, G_{D1}\right) = b_2 \frac{\alpha_{31}}{\beta_{31}} \Bigg[& G_{T3} \frac{TSH}{TSH + D_T} \\ & + G_{D1} \left(\frac{T_{4,th} \frac{TSH}{TSH+k}}{K_{M1} + T_{4,th} \frac{TSH}{TSH+k}} + \frac{FT_4}{FT_4 + K_{M1}} \right) \\ & + G_{D2} \left(\frac{T_{4,th} \frac{TSH}{TSH+k}}{K_{M2} + T_{4,th} \frac{TSH}{TSH+k}} + \frac{FT_4}{FT_4 + K_{M2}} \right) \Bigg] \end{aligned}$$

where

$$b_2 = \frac{1}{1 + K_{30} TBG}$$
$$T_{4,th} = G_T \frac{TSH}{TSH + D_T}$$

It can be seen that $FT_{3,eq}\left(G_{T3}, k, G_{D1}\right)$ is affine in G_{D1} and G_{T3} for a fixed value of k and given the equilibrium concentrations of TSH and T_4, which can be computed independently of G_{D1} and G_{T3}. This fact is used in the parameter identification in the main article.

In the dynamic simulations of the model shown in Section 3 of the main text, we observe that the stationary hormone levels are slightly higher than the equilibrium value $FT_{3,eq}$ computed above. This is due to the following. For our dynamic simulation, the TRH concentration arriving at the pituitary has a circadian oscillation with additional stochastic noise with a log-normal distribution, as discussed in detail in [3]. On the other hand, for the parameter identification described in Section 2 of the main text, a constant (equilibrium) value for TRH was assumed. The presence of the additional log-normally distributed noise causes an offset in TSH (its mean value increases by approximately 17%) which in turn results in the observed slight increase in FT_3 levels. The transient increase in FT_3-levels, which can be observed in both plots in Figure 3 of the main text, is a result of this additional noise and the chosen initial conditions for the simulation.

S2 FORMAL DEFINITION OF SENSITIVITY ANALYSIS

The purpose of this section is to formally define the first-order sensitivity matrix of a system of ordinary differential equations (ODEs) w.r.t. several parameters, following the exposition in [4]. As stated in Section 4 in the main article, we consider ODEs of the form

$$\dot{x}(t) = f(t, x, p), \quad x(t_0) = x_0. \tag{S10}$$

where

- $t \in I$ denotes time, where I is some closed interval $I = [t_0, t_1]$ on which the solution x to (S10) is defined
- $p \in \mathbb{R}^m$ denotes the m-dimensional parameter vector
- $x : I \times \mathbb{R}^m \to \mathbb{R}^n$ denotes the solution to (S10), i.e. $x(t, p)$ is the value of the system state at time t for a given parameter p
- $x_0 \in \mathbb{R}^n$ is the system's initial value
- $f : I \times \mathbb{R}^n \times \mathbb{R}^m \to \mathbb{R}^n$ is a continuous vector field that is continuously differentiable w.r.t. x and p

It can be shown (cf. [4]) that if the system (S10) has a unique solution $x(t, p_0)$ over I for some fixed (nominal) parameter vector p_0, then it also has a unique solution $x(t, p)$ for all parameter variations p sufficiently close to p_0, i.e. for all p such that $\|p - p_0\|$ is sufficiently small. Thus, we can define the sensitivity matrix $S : I \to \mathbb{R}^{n \times m}$ as the matrix of partial derivatives of the components of x w.r.t. to the components of p:

$$(S(t))_{ij} = \left. \frac{\partial x_i(t, p)}{\partial p_j} \right|_{(t, p_0)} \tag{S11}$$

Note that, indeed, this matrix describes how sensitive each state component is w.r.t. variations in one component of the parameter vector p around some nominal value. According to [4], $S(t)$ can be computed by solving the following system of ODEs:

$$\begin{aligned} &\dot{x}(t) = f(t, x, p_0) \\ &\dot{S}(t) = \left[\frac{\partial f(t, x, p)}{\partial x}\right]_{p=p_0} \cdot S + \left[\frac{\partial f(t, x, p)}{\partial p}\right]_{p=p_0} \\ &x(t_0) = x_0, \quad S(t_0) = 0 \end{aligned} \tag{S12}$$

This approach can easily be extended to time-varying parameter vectors which is needed in the modeling of the pituitary-thyroid feedback loop due to the sinusoidal circadian rhythm of TRH.

S3 NUMERICAL PARAMETER VALUES FOR THE IMPLEMENTATION OF THE PRESENTED MODEL

Symbol	Description	Value	Origin
TBG	Concentration of thyroxine-binding Globulin	$300\,nmol/l$	[5] / well-known reference value
$TBPA$	Concentration of Transthyretin	$4.5\,\mu mol/l$	[5] / well-known reference value
IBS	Concentration of intra-cellular T_3-binding substrate	$8\,\mu mol/l$	Estimated from TBG-level, corrected for intra-cellular T_3-accumulation (according to values from [6])
TRH	TRH-level in hypophyseal portal system	$6.9\,nmol/l$	[7]
G_H	Secretory capacitiy of the pituitary	$817\,mU/s$	Calculated according to values from [8] and [9]
D_H	Damping constant (EC_{50}) of TRH at the pituitary	$47\,nmol/l$	[10]
α_S	Dilution factor for peripheral TSH	$0.4\,l^{-1}$	Reciprocal value of the volume of distribution for plasma volume of $2.5\,l$
β_S	Clearance exponent for peripheral TSH	$2.3 \cdot 10^{-4} s^{-1}$	Calculated from plasma half-life of 50 min ([11, 12])
L_S	Brake constant of long feedback	$1.68\,l/\mu mol$	Calculated from clinical data of hyperthyroid patients
G_T	Secretory capacity of thyroid gland	$3.4\,pmol/s$	[12]
D_T	Damping constant (EC_{50}) of TSH at the thyroid gland	$2.75\,mU/l$	[13]

Table S1. Numerical parameter values for the implementation of the presented model of the HPT axis, adopted from [14] - part 1.

Symbol	Description	Value	Origin
α_T	Dilution factor for T_4	$0.1\, l^{-1}$	Reciprocal value of the volume of distribution (the latter from [15])
β_T	Clearance exponent for T_4	$1.1 \cdot 10^{-6} s^{-1}$	Calculated from plasma half-life of 7 days ([16, 15])
K_{M1}	Dissociation constant of 5'-deiodinase I	$500 nmol/l$	[15]
α_{31}	Dilution factor for peripheral T_3	$2.6 \cdot 10^{-2} l^{-1}$	Reciprocal value of the volume of distribution (the latter from [15])
β_{31}	Clearance exponent for peripheral T_3	$8 \cdot 10^{-6} s^{-1}$	Calculated from plasma half-life of 24 h ([15])
G_{D2}	Maximum activity of type II deiodinase	$4.3\, fmol/s$	Calculated from pituitary T_3-level ([17])
K_{M2}	Dissociation constant of 5'-deiodinase II	$1\, nmol/l$	[18]
α_{32}	Dilution factor for central T_3	$1.3 \cdot 10^{-5} l^{-1}$	Calculated from volume of distribution $7.6 \mu l$
β_{32}	Clearance-Exponent for central T_3	$8.3 \cdot 10^{-4} s^{-1}$	Calculated from intra-cellular half-life of 15 min [19] and [20])
α_{S2}	Dilution factor for pituitary TSH	$2.6 \cdot 10^{-5} l^{-1}$	Calculated from volume of distribution $3.8 \mu l$
β_{S2}	Clearance exponent for pituitary TSH	$140\, s^{-1}$	estimated corresponding to half-life of $5s$
D_R	Damping constant for central T_3	$100\, pmol/l$	[21]
G_R	Maximum gain of TRβ receptors	$1\, mol/s$	Value unknown, normalized to 1 (magnitude of feedback is determined by L_S)
S_S	Brake constant of ultrashort feedback	$100\, l/mU$	Determined according to values from [22]
D_S	Damping constant for TSH inside the pituitary	$50\, mU/l$	Determined according to values from [22]

Table S2. Numerical parameter values for the implementation of the presented model of the HPT axis, adopted from [14] - part 2.

Symbol	Description	Value	Origin
K_{30}	Dissociation constant T_3-TBG	$2 \cdot 10^9 l/mol$	[12]
K_{31}	Dissociation constant T_3-IBS	$2 \cdot 10^9 l/mol$	Value unknown, adapted to extra-cellular dissociation constant
K_{41}	Dissociation constant T_4-TBG	$2 \cdot 10^{10} l/mol$	[12]
K_{42}	Dissociation constant T_4-$TBPA$	$2 \cdot 10^8 l/mol$	[12]
τ_{0S}	Peripheral delay for TSH	$120\ s$	Derived from circulation time
τ_{0S2}	Pituitary delay for TSH	$3240\ s$	Derived from period of TSH-pulses (data from [23, 24])
τ_{0T}	Delay for T_4	$300\ s$	Estimated according to circulation and diffusion times
τ_{03Z}	Delay for pituitary T_3	$3600 s$	Derived from [25]

Table S3. Numerical parameter values for the implementation of the presented model of the HPT axis, adopted from [14] - part 3.

Model Configuration	Parameter	Value
(1) no shunt	G_{T3}	-
	G_{D1}	$27.5\, nmol/s$
	k	-
(2) full TSH-T_3-shunt	G_{T3}	$394\, fmol/s$
	G_{D1}	$22\, nmol/s$
	k	$1\, mU/l$

Table S4. Numerical parameter values for the implementation of the presented model of the HPT axis - parameters identified via least squares estimation. G_{T3} is the gain of the Michaelis-Menten-Hill kinetics in the TSH-T_3-Shunt, G_{D1} describes the maximum activity of type I deiodinase. The parameter k of the Michaelis-Menten-Hill kinetics that is used to model the TSH-stimulated deiodination inside the shunt was normalized to $1\frac{mU}{l}$. There are two different model configurations for which we separately identified the parameters: The model from [14] without the TSH-T_3-shunt (1), and the model including the full shunt as described in the main article (2). For version (2), the optimal parameters cannot be determined uniquely. In this case, the above values correspond to a peripheral contribution to the T_3 production of approximately 80%, as described in Section 2 in the article.

REFERENCES

[1]J. D. Murray. *Mathematical Biology I: An Introduction*. Springer, 3. edition, 2002.

[2]F. Keller and D. Zellner. The 1-exp function as an alternative model of non-linear saturable kinetics. *Eur J Clin Chem Clin Biochem*, 34:265–271, 1996.

[3]J. W. Dietrich, A. Tesche, C. R. Pickardt, and U. Mitzdorf. Thyrotropic feedback control: Evidence for an additional ultrashort feedback loop from fractal analysis. *Cybernetics and Systems*, 35(4):315–31, 2004. DOT:10.1080/01969720490443354.

[4]H. Khalil. *Nonlinear Systems*. Macmillan, 2001.

[5]D. Neubert. Schilddrüsenhormon. *Endokrinologie II*, 19:65–212, 1977.

[6]M. T. Hays, M. R. Broome, and J. M. Turrel. A multicompartmental model for iodide, thyroxine, and triiodothyronine metabolism in normal and spontaneously hyperthyroid cats. *Endocrinology*, 122(6):2444–61, 1988.

[7]J. M. M. Rondeel, W. J. de Greef, P. van der Schoot, B. Karels, W. Klootwijk, et al. Effect of the thyroid status and paraventricular area lesions on the release of thyrotropin-releasing hormone and catecholamines into hypophysial portal blood. *Endocrinology*, 123:523–7, 1988.

[8]S. A. D'Angelo, D. H. Paul, N. R. Wall, and D. M. Lombardi. Pituitary thyrotropin (TSH) rebound phenomenon and kinetics of secretion in the goitrous rat: differential effects of thyroxine on synthesis and release of TSH. *Endocrinology*, 99(4):935–43, 1976.

[9]A. Okuno, T. Taguchi, K. Nakayama, and M. Takamoto. Kinetic analysis of plasma TSH dynamics after TRH stimulation. *Horm. Metab. Res.*, 11:293–5, 1979.

[10]M. Le Dafniet, A.-M. Brandi, M. Kujas, P. Chanson, and F. Peillon. Thyrotropin-releasing hormone (TRH) binding sites and thyrotropin response to TRH are regulated by thyroid hormones in human thyrotropic adenomas. *European Journal of Endocrinology*, 130:559–64, 1994.

[11]W. D. Odell, R. D. Utiger, J. F. Wilber, et al. Estimation of the secretion rate of thyrotropin in man. *Journal of Clinical Investigation*, 46(6):953–9, 1967.

[12]G. Li, B. Liu, and Y. Liu. A dynamical model of the pulsatile secretion of the hypothalamo-pituitary-thyroid axis. *Biosystems*, 35(1):83–92, 1995.

[13]J. E. Dumont and G. Vassart. Thyroid regulation. *Endocrinology DeGroot, WB Saunders*, 1995.

[14]J. W. Dietrich. *Der Hypophysen-Schilddrüsen-Regelkreis: Entwicklung und klinische Anwendung eines nichtlinearen Modells*. Spektrum medizinischer Forschung, ed. F. Schardt. Vol. 2 (Berlin: Logos-Verlag), 2002.

[15]F. S. Greenspan. *Basic & Clinical Endocrinology*, chapter The Thyroid Gland, pages 192–262. Stamford, CT, Appleton & Lange, 1997.

[16]M. Grußendorf. *Metabolismus der Schilddrüsenhormone*. 1°, Stuttgart, New York, Thieme, 1988.

[17]J. van Doorn, F. Roelfsema, and D. van der Heide. Concentrations of thyroxine and 3,5,3'-triiodothyronine at 34 different sites in euthyroid rats as determined by an isotopic equilibrium technique. *Endocrinology*, 117(3):1201–8, 1985.

[18]T. J. Visser, M. M. Kaplan, J. L. Leonard, and P. R. Larsen. Evidence for two pathways of iodothyronine 5'-deiodination in rat pituitary that differ in kinetics, propylthiouracil sensitivity, and response to hypothyroidism. *J. Clin. Invest.*, 71(4):992–1002, 1983.

[19]J. H. Oppenheimer, G. Bernstein, and J. Hasen. Estimation of rapidly exchangeable cellular thyroxine from the plasma disappearance curves of simultaneously administered thyroxine-131i and albumin 125i. *Journal of Clinical Investigation*, 46(5):762–77, 1967.

[20]S. Benvenga and J. Robbins. Thyroid hormone efflux from monolayer cultures of human fibroblasts and hepatocytes. effect of lipoproteins and other thyroxine transport proteins. *Endocrinology*, 139(10):4311–8, 1998.

[21]M. A. Lazar and W. W. Chin. Nuclear thyroid hormone receptors. *J. Clin. Invest.*, 86:1777–82, December 1990.

[22]T. Kakita, N.P. Laborde, and W.D. Odell. Autoregulatory control of thyrotropin in rabbits. *Endocrinology*, 114(6):2301–5, 1984.

[23]S. L. Greenspan, A. Klibanski, D. Schoenfeld, et al. Pulsatile secretion of thyrotropin in man. *Journal of Clinical Endocrinology and Metabolism*, 63(3):661–8, 1986.

[24]G. Brabant, U. Ranft, K. Ocran, et al. Thyrotropin - an episodically secreted hormone. *Acta-Endocrinol,Copenh*, 112(3):315–22, 1986.

[25]P. R. Larsen. Feedback regulation of thyrotropin secretion by thyroid hormones. *N Engl J Med*, 306(1):23–32, 1982.
